# Timelines and Associated Factors for Return-to-Work of Patients With Painful Lumbar Radiculopathy Who Undergo Lumbar Microdiscectomy Followed by Physiotherapy: A Prospective Cohort Study

**DOI:** 10.1097/BRS.0000000000005443

**Published:** 2025-07-14

**Authors:** Servan Rooker, Stijn J. Willems, Niels Franken, Martijn W. Heymans, Michel W. Coppieters, Martijn S. Stenneberg, Gwendolyne G.M. Scholten-Peeters

**Affiliations:** a Department of Family Medicine and Population Health (FAMPOP), University of Antwerp, Antwerp, Belgium; b Department of Neurosurgery, Kliniek ViaSana, Mill, The Netherlands; c Faculty of Behavioural and Movement Sciences, Vrije Universiteit Amsterdam, Amsterdam Movement Sciences, Program Musculoskeletal Health, Amsterdam, The Netherlands; d SOMT University of Physiotherapy, Amersfoort, The Netherlands; e Department of Epidemiology and Biostatistics, VU Medical Center, Amsterdam, The Netherlands; f School of Health Sciences and Social Work, Griffith University, Brisbane and Gold Coast, Australia; g Department of Physiotherapy, Human Physiology and Anatomy (KIMA), Experimental Anatomy Research Group, Vrije Universiteit Brussel, Belgium

**Keywords:** return-to-work, sciatica, occupational health, rehabilitation, neurosurgery, prognosis, surgery

## Abstract

**Study Design.:**

Prospective cohort study with a 52 weeks follow-up.

**Objective.:**

Medical absenteeism in patients with painful lumbar radiculopathy undergoing lumbar microdiscectomy followed by physiotherapy is associated with high socioeconomic costs. We lack good quality information about the time to return-to-work and the factors associated with returning-to-work in this patient group. The objective of this study is to describe the probability of return-to-work and explore associations between routinely collected preoperative factors and return-to-work for patients with painful lumbar radiculopathy undergoing lumbar microdiscectomy and postoperative physiotherapy.

**Materials and Methods.:**

We included 257 patients with clinical signs and symptoms of painful lumbar radiculopathy in whom nerve root compression was confirmed by magnetic resonance imaging, and who underwent microdiscectomy and postoperative physiotherapy. Time to return-to-work was evaluated using Kaplan-Meier survival analysis. The association between independent factors and return-to-work was examined through Cox regression analysis.

**Results.:**

Full resumption of their original paid job (*i.e.* same role with the same physical demands and responsibilities) occurred in 178 (69.3%) of participants by 52 weeks. In these patients, the median (IQR) return-to-work time was 16 weeks (14–16), with 85.0% of patients resuming work within 26 weeks. Higher education (HR=1.82), self-employment (HR=1.84), and the absence of predominant physical work (HR=1.61) were significantly associated with a faster return-to-work, while higher disability scores negatively impacted return-to-work time (HR=0.56).

**Conclusion.:**

At 52 weeks following lumbar microdiscectomy and postoperative physiotherapy for painful lumbar radiculopathy, approximately two-thirds of individuals returned to work in their original roles, while some transitioned to different roles. Work-related and personal factors play a key role in determining the timing of this return. Recognizing these predictors in clinical practice can help surgeons, physiotherapists, and occupational health professionals guide patient expectations, provide more individualized workplace counselling, and support realistic, timely, and sustainable work reintegration.

Returning-to-work is an important outcome measure for patients with painful lumbar radiculopathy undergoing lumbar microdiscectomy and postoperative physiotherapy, as work absenteeism is associated with substantial socioeconomic costs.^1,2^ Of patients who undergo lumbar microdiscectomy, 5% to 22% do not return-to-work for up to 24 months.^3–8^ In addition, long-term follow-up studies show that 17.0% to 19.4% of patients either do not return-to-work or do not return to their previous work capacity within 5 to 20 years.^9–11^

Multiple factors have been associated with poor return-to-work time in patients undergoing surgery for lumbar radiculopathy.^12–15^ Systematic reviews have identified preoperative pain and disability levels, timing before surgery, symptoms of depression, occupational mental stress, lateral disc prolapse, and the duration of sick leave before surgery as significant contributors.^12–16^ In addition, a lack of job satisfaction has been consistently linked to unfavorable return-to-work rates, while the female sex was associated with an 11% higher risk of failing to return-to-work after microdiscectomy.^12–14^ However, conducting meta-analyses for many other variables was challenging as most factors were either reported in single studies or had incomplete data.^14,15^ Moreover, the current literature on this topic has several methodological limitations. Many studies included in systematic reviews suffer from a high risk of bias, often due to inappropriate outcome measures, small sample sizes, substantial loss to follow-up, and the use of unadjusted models.^12,14,17^ Furthermore, many existing studies include heterogeneous surgical techniques, such as lumbar fusion and chemonucleolysis, that are not typically recommended for isolated lumbar radiculopathy.^12,18^ This heterogeneity makes it difficult to isolate the specific impact of lumbar microdiscectomy on return-to-work outcomes. Despite the clear clinical and socioeconomic relevance of this outcome, there remains a significant lack of recently published high-quality prospective cohort studies focused exclusively on return-to-work following lumbar microdiscectomy. Findings from these studies provide valuable insights into return-to-work timelines and associated factors, enabling clinicians to help patients set realistic goals, establish achievable expectations, and support their recovery and return to preoperative function and employment.

The primary aim of this study was to describe the probability of return-to-work in patients with painful lumbar radiculopathy undergoing lumbar microdiscectomy followed by physiotherapy. The secondary aim was to explore associations between routinely collected preoperative factors and return-to-work outcomes, with a follow-up period of 52 weeks.

## MATERIALS AND METHODS

### Design

We conducted a prospective cohort study with 52 weeks of follow-up. The study adhered to the recommendations for reporting cohort studies as outlined in the Strengthening the Reporting of Observational Studies in Epidemiology (STROBE) statement. Follow-up data were collected using standardized patient-reported outcome measures.^19^

### Study Population

Patients were recruited from the ViaSana Clinic, a multidisciplinary hospital in Mill, The Netherlands. Consecutive patients older than 18 years with (1) signs and symptoms from patient history and physical examination indicative of lumbosacral nerve root compression; (2) Magnetic Resonance Imaging (MRI) confirmed nerve root compression corresponding with clinical signs and symptoms; and (3) an indication for lumbar microdiscectomy, were eligible to participate in this study. Patients were excluded in case of serious pathology, such as tumors, fractures, cauda equina syndrome, those with a history of laminectomy, currently pregnant, insufficient proficiency with the Dutch language or not having paid work before surgery.

All patients underwent lumbar microdiscectomy performed by either an orthopedic surgeon or a neurosurgeon, both with over 10 years of experience. The orthopedic and neurosurgeon were members of the same surgical unit and used comparable surgical techniques. The procedure involved relieving pressure on the affected lumbar nerve root by removing part of the intervertebral disc and ligamentum flavum, using a surgical microscope. Postoperative imaging was not routinely performed unless clinically indicated (*e.g.* persistent symptoms or suspected complications).

All patients received one in-hospital physiotherapy session the day after surgery, which included education on postoperative recovery, home care instructions, and mobility exercises. Upon discharge, patients were referred to a primary care physiotherapist with a standardized rehabilitation protocol (Appendix A, Supplemental Digital Content 1, http://links.lww.com/BRS/C766).^20^ This protocol emphasized restoring mobility, trunk muscle training, and physical endurance, and included guidance on resuming walking, cycling, and work-related activities. While the core treatment plan was standardized, it was adapted to individual factors, such as treatment goals, job demands, and fitness levels. Patients were encouraged to resume work and physical activity as soon as feasible, ideally within 26 weeks postsurgery.

### Data Collection

Data were collected using OnlinePROMS (Interactive Studios, Rosmalen, The Netherlands), a program to manage, send, and process, questionnaires (http://www.onlineproms.nl/). Baseline assessments were conducted preoperatively using routinely collected data. Patients who did not respond or only partially responded to the digital and mailed invitations were contacted by telephone up to three times to encourage completion of the questionnaires.

### Outcome Measurement

The primary outcome of interest was time until full return-to-work following lumbar microdiscectomy and subsequent physiotherapy. This outcome was derived from a questionnaire that assessed the current employment status (“Do you have paid work currently?”) and the number of weeks until full work resumption (“After how many weeks did you fully resume your work?”). Alongside, the nature of the return-to-work was documented (*i.e.* “Did you fully resume your job with the same tasks as before the surgery?”). Full return-to-work was defined as patients who, at 52 weeks postsurgery, were engaged in paid employment and had resumed their presurgery job without any modifications or restrictions to their tasks. This served as the primary event of interest. Patients who remained on sick leave beyond 52 weeks postsurgery, or who returned to work in a different role or with restrictions, were classified as having “not fully returned to work” (*i.e.* did not fully resume their presurgery job).

### Candidate Factors Associated With Return-to-Work

Candidate factors for return-to-work were selected based on existing literature and their potential to be routinely collected in clinical settings, ensuring their relevance and practicality for clinical practice.^12–14,21–23^ Factors were categorized into five domains and included: (1) Sociodemographic factors: sex, age, educational level, and comorbidity; (2) Clinical factors: preoperative back and leg pain intensity (visual analog scale 0–100  mm), distribution between back and leg pain (VAS), baseline disability (Roland Disability Questionnaire 0–24 points); (3) Presurgical treatment factors: prior lumbar microdiscectomy, prior physiotherapy, pain medication preoperative, injection therapy preoperative; (4) Medical imaging findings: level of disc herniation, comorbidity seen on MRI (such as degeneration, arthrosis, cyst); (5) Work-related factors: payment type (employee, self-employed), physical work demands (predominantly physical work *vs.* nonphysical), and sitting work demands (predominantly sitting *vs.* nonsitting). The categorical variables were defined as “absent *versus* present,” “yes or no,” or “positive or negative.” The Roland Disability Questionnaire was dichotomized into high *versus* low disability levels, using a cutoff point of 17/24 points for high disability.^24–26^

### Selection of Factors Associated With Return-to-Work

The selection of relevant factors from the candidate factors for analysis and confounding variables was determined by consensus from a clinical expert panel (n=5), comprising one neurosurgeon, two orthopedic surgeons, and two physiotherapists in collaboration with a patient representative. The factors of educational level, physical work demands, employment status, and disability level were selected based on their assumed association with return-to-work rates and prior research demonstrating their effect on recovery outcomes following lumbar surgeries.^12–14,21–23^ For example, lower education and higher disability are consistently linked to delayed recovery and prolonged disability.^12–14,21–23^ The expert group theorized that work-related factors, including physical work demands and employment status, play a crucial role in return-to-work outcomes. High physical demands tend to delay recovery, while self-employed individuals often return-to-work sooner due to financial incentives. The variables age, sex, and baseline pain scores were included as potential confounders to adjust for their influence on recovery outcomes.^12–14,21–23^ Age was included as recovery rates often vary across age groups; sex was adjusted for due to biological differences that may affect postsurgical recovery; and baseline pain scores were accounted for, given their status as a primary indication for surgery and their known influence on recovery outcomes.^12–14,21–23^

### Sample Size Calculation

A sample size calculation was performed to ensure sufficient power for detecting significant associations in the Cox proportional hazards model.^27,28^ The analysis targeted an 80% power, a significance level of 5% (α=0.05), and an assumed hazard ratio (HR) of two for the primary covariate of interest. To account for the model’s complexity, a corrected HR representing the combined effect of multiple covariates was utilized.

Simulations indicated that ∼236 events (instances of return-to-work) would be required to achieve the desired statistical power. With an anticipated sample size of 250 participants, this approach ensures that the study is adequately powered to detect meaningful associations between the covariates and return-to-work outcomes.

### Statistical Analysis

Descriptive statistics were used to present patient characteristics. The baseline data were checked for normality through the Shapiro-Wilk test, histograms, and quantile-quantile (Q-Q) plots. Continuous data that were normally distributed are described by means and SDs. In case of violation of normality, medians and interquartile ranges (IQR) are presented.

The Kaplan-Meier survival analysis was used to investigate the duration until full return-to-work in weeks.^29^ Univariable and multivariable Cox proportional hazard regression models were applied to calculate hazard ratios (HRs) and their 95% CIs for relevant variables, with adjustments made for the confounders age, sex, and leg pain intensity in both models. This approach allowed us to assess each factor’s independent effect in the univariable models and to evaluate its effect adjusted for all selected variables and confounders in the multivariable models. A complete case analysis was performed. The proportional hazards assumption was tested using scaled Schoenfeld residuals. Kaplan-Meier curves were used to visually depict survival probabilities for return-to-work for significant factors identified by the multivariable Cox model. All analyses were performed using the “survival,” “survminer,” and “powerSurvEpi” packages in R statistical software, version 3.5.1.

## RESULTS

### Study Population

**Figure 1:**
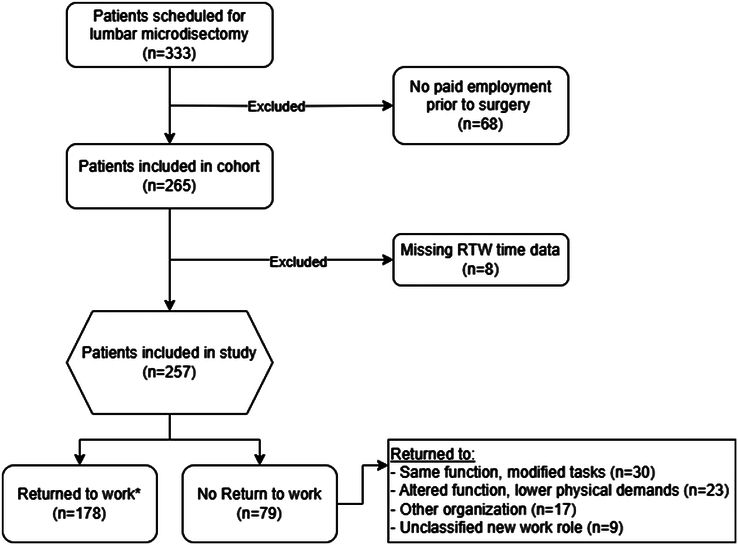
Participants flowchart. *RTW is defined as the resumption of paid employment with the same job function, tasks, and physical work demands, without restrictions or modifications. RTW indicates return-to-work.

The study population initially consisted of 333 patients. After excluding 68 individuals who were not in paid employment before surgery, 265 patients met the eligibility criteria for inclusion. Of these, eight patients were excluded due to missing return-to-work data, resulting in a final study population of 257 patients. Of the 257 included patients, approximately half underwent surgery performed by an orthopedic surgeon and the other half by a neurosurgeon. Figure 1 shows the patient’s flow diagram, and Table 1 summarizes the baseline characteristics. The mean age was 43.3 years (SD: 10.1), and 157 patients (61.1%) were male. The median leg pain score (VAS) was 75.0 (IQR: 55.4–87.1). Thirty-eight patients had undergone prior lumbar microdiscectomy, with a median interval of seven years between the initial and current surgery (IQR: 5.4–9.1 yr). Among these, 19 patients (50%) had microdiscectomy at the same spinal level, and 18 patients (47%) had surgery on the same side. On the basis of available follow-up data, 240 of 257 patients (93.4%) received postoperative physiotherapy. The median duration was 14 weeks (IQR: 8–26 wk; range: 1–52 wk). Most patients received core components of care consistent with postoperative guidelines, including education (91%), therapeutic exercises (89%), and mobility training (81%).

**TABLE 1:** Patient Characteristics (n=257)

Sociodemographic	
Age (mean ±SD) in years	43.3±10.1
Male gender, n (%)	157 (61.1)
Comorbidity*, n (%)	46 (18.0)
Educational level (high), n (%)	79 (30.9)
Body mass index (mean±SD)	25.57±3.57
History, n (%)	
Prior lumbar microdiscectomy†	38 (14.8)
Previous physiotherapy	191 (74.3)
Previous injection therapy	41 (16.0)
Preoperative pain medication use	134 (52.1)
Symptoms/functioning	
Intensity back pain (VAS)‡ [median (IQR)]	49.8 (20.0–74.0)
Intensity leg pain (VAS)‡ [median (IQR)]	75.0 (55.4–87.1)
Leg pain more than back pain, n (%)	184 (72.8)
Level of disability (RMQ)§ [median (IQR)]	17.0 (14.0–20.0)
Radiologic findings	
Level of disc herniation, n (%)	
L3–L4	9 (3.5)
L4–L5	85 (33.1)
L5–S1	139 (54.1)
More than one level, n (%)	20 (7.8)
Structural changes on MRI at the affected level of disc herniation‖	99 (38.5)
Work-related characteristics, n (%)	
Predominantly physically work	104 (40.5)
Predominantly sedentary work	85 (33.1)
Salaried employment	220 (85.6)

* Comorbidity (*e.g.* cardiovascular disease, chronic obstructive pulmonary disease, hyperthyroidism).

† All prior surgeries involved lumbar microdiscectomy at the same or a different spinal level.

‡ VAS: visual analog scale (0–100 mm).

§ RDQ: Roland-Morris Disability Questionnaire (0–24 points).

‖ Structural changes on MRI at the affected level of disc herniation, spinal stenosis, spinal cyst, facet arthrosis, hypoplastic disc, or a combination

IQR indicates interquartile range.

### Return-to-Work

**Figure 2:**
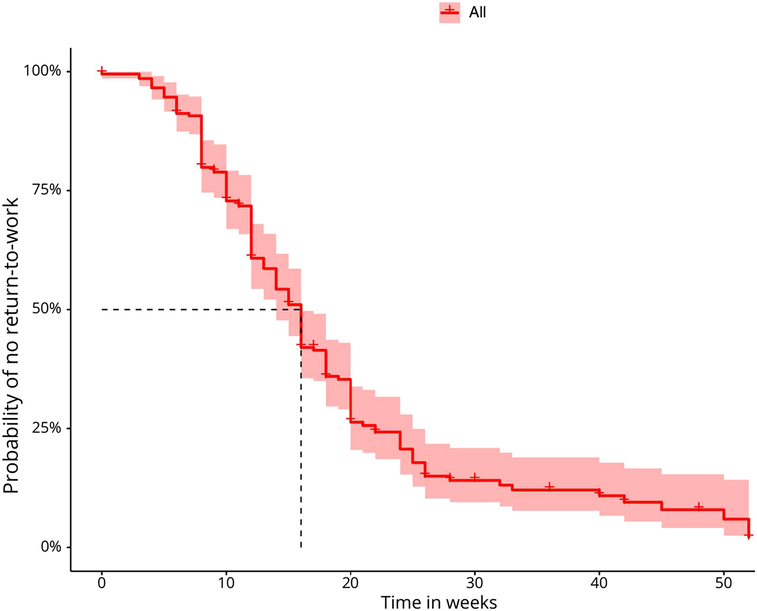
Probability of remaining out of work (no return-to-work) over time (in weeks) after lumbar microdiscectomy among patients who returned to work (n=178), with CIs. The median return-to-work time is indicated by a dotted line in the graph.

By 52 weeks, 178 patients (69.3%) had fully resumed their paid job in their preoperative role, maintaining the same tasks and physical demands, and were classified as having fully returned to work following lumbar microdiscectomy and physiotherapy for painful radiculopathy. Among these patients, the median (IQR) return-to-work time was 16.0 weeks (14.0–16.0), with an 85% probability of returning-to-work within the first 26 weeks postsurgery (Figure 2). In addition, 23 patients (9.0%) transitioned to a less physically demanding role, 30 patients (11.7%) continued in the same role but with modified tasks, and 17 patients (6.6%) took on a job with similar physical demands but a different job description. The work roles of nine patients (3.4%) remained unclassified.

### Univariable Association With Return-to-Work

The univariable Cox regression analysis identified significant associations between several factors and return-to-work times (Table 2). Higher educational levels were linked to a faster return-to-work, with a crude HR of 1.84 (95% CI: 1.33–2.55, *P*<0.001). Similarly, individuals with lower physical work demands returned to work more quickly, showing a crude HR of 1.78 (95% CI: 1.28–2.47, *P*=0.01). In contrast, higher disability scores were associated with slower return-to-work times, with a crude HR of 0.60 (95% CI: 0.44–0.83, *P*<0.001). Although self-employed individuals showed a trend toward faster return-to-work (crude HR of 1.38, 95% CI: 0.89–2.13), this was not statistically significant (*P*=0.147) (Table 2; Appendix B, Supplemental Digital Content 2, http://links.lww.com/BRS/C767). Adjusting for confounders did not significantly change these associations (Table 2; Appendix B, Supplemental Digital Content 2, http://links.lww.com/BRS/C767).

**TABLE 2:** Hazard Ratios for the Univariable Association of the Individual Factors With Return-to-Work: Crude and Adjusted for Confounders

	Univariable association with return-to-work			
	Crude HR (95% CI)	*P*	Adjusted HR (95% CI)*	*P* *
Educational level (high)	1.84 (95% CI: 1.33–2.55)	<0.001	1.92 (95% CI: 1.38–2.68)	<0.001
Self-employed (yes)	1.38 (95% CI: 0.89–2.13)	0.147	1.34 (95% CI: 0.86–2.08)	0.189
Disability score (high)	0.60 (95% CI: 0.44–0.83)	<0.001	0.54 (95% CI: 0.38–0.76)	<0.001
Physical work (no)	1.78 (95% CI: 1.28–2.47)	0.01	1.79 (95% CI: 1.29–2.50)	0.01

High disability defined as >17/24.

* Adjusted for the confounders: age, sex, and leg pain intensity.

HR indicates hazard ratio.

### Multivariable Association With the Return-to-Work

**Figure 3:**
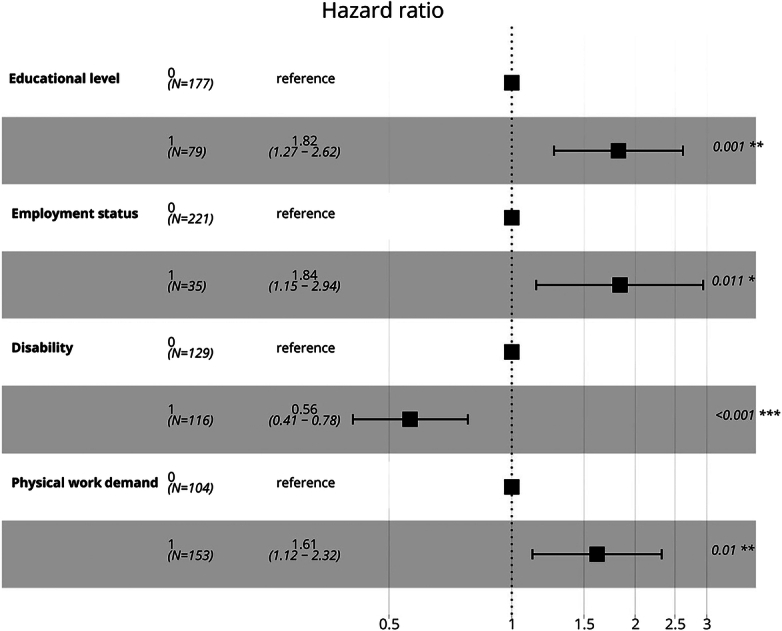
Forest plot visualizing the crude multivariable cox regression model.

**Figure 4:**
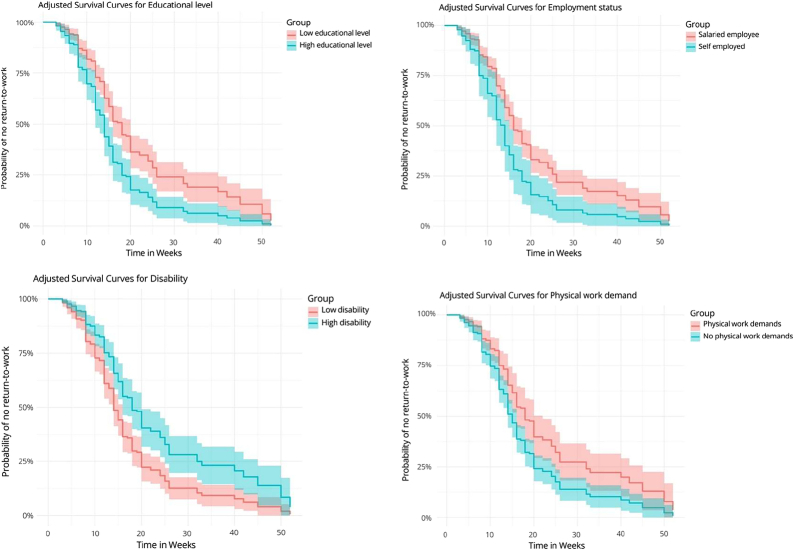
Kaplan-Meier survival curves illustrating significant no return-to-work probability.

The Cox regression analysis indicated that, when examined together, a high level of education and self-employment status were significantly linked to faster return-to-work times, with HR’s of 1.82 (95% CI: 1.27–2.62, *P*<0.001) and 1.84 (95% CI: 1.15–2.94, *P*=0.01), respectively. Furthermore, the absence of predominant physical work was significantly associated with a faster return-to-work time, with an HR of 1.61 (95% CI: 1.12–2.32, *P*=0.01). Conversely, a high disability score was associated with a decreased return-to-work time, with an HR of 0.56 (95% CI: 0.41–0.78, *P*<0.001), highlighting the negative impact of severe disability on the ability to return-to-work (Table 3, Figure 3). Adding the confounders to the crude model did not significantly influence the overall outcomes (Table 3; Appendix C, Supplemental Digital Content 3, http://links.lww.com/BRS/C768). Survival probabilities are illustrated using Kaplan-Meier curves in Figure 4.

**TABLE 3:** Hazard Ratios for the Multivariable Association of Combined Factors With Return-to-Work: Crude and Adjusted Models for Confouding

	Multivariable association with return-to-work			
	Crude HR (95% CI)	*P*	Adjusted HR (95%CI)*	*P* *
Educational level (high)	1.82 (95% CI: 1.27–2.62)	<0.001	2.01 (95% CI: 1.37–2.95)	<0.001
Self-Employed (yes)	1.84 (95% CI: 1.15–2.94)	0.01	1.73 (95% CI: 1.08–2.78)	0.01
Disability score (high)	0.56 (95% CI: 0.41–0.78)	<0.001	0.50 (95% CI: 0.35–0.71)	<0.001
Physical work (no)	1.61 (95% CI: 1.12–2.32)	0.01	1.49 (95% CI: 1.49–2.81)	0.01

High disability defined as >17/24.

* Adjusted for the confounders: age, sex, and leg pain intensity.

HR indicates hazard ratio.

## DISCUSSION

This study aimed to describe the probability of returning-to-work after lumbar microdiscectomy, followed by physiotherapy, and to identify factors associated with return-to-work. Two-thirds of the patients fully resumed their paid job in their original role with the same physical demands and responsibilities by 52 weeks. The median return-to-work time for these patients was 16.0 weeks (IQR: 14.0–16.0 wk), with an 85% probability of returning-to-work within the first 26 weeks. However, some patients transitioned to less physically demanding positions or modified their tasks to accommodate their recovery. Higher education, self-employment, and the absence of predominant physical work were significantly associated with an increased chance of returning-to-work, while high disability scores negatively impacted return-to-work time. Understanding these associations may assist surgeons, physiotherapists, and occupational health professionals in guiding patient expectations, providing more personalized counseling, and supporting realistic, timely, and sustainable return-to-work planning.

The findings of this study are in line with previous cohort studies, which report that an estimated 62% to 95% of patients returning to full-time work within two years after microdiscectomy.^3–11,30,31^ The relatively wide range in the literature can be explained by the varying definitions of return-to-work, the sample size, the inclusion of different patient groups, and differences in employment type, which has been shown to influence return-to-work timelines ranging from five to more than 16 weeks.^14,32–34^ Interestingly, only two-thirds of the patients in our study fully returned to their jobs, performing the same tasks and responsibilities as before their surgery. This aligns with other studies that report a range of return-to-work outcomes, where a significant portion of patients resumed work in modified roles or with adjusted tasks to accommodate postoperative limitations.^10,12,30,33^

Although employment status was not a significant factor in the univariable analysis, it became significant in the multivariable model. This suggests that the factors of educational level, physical work demands, and disability score likely influenced its effect in combination. Self-employed individuals with high educational level, low physical work demands, and low disability scores tend to return-to-work faster, possibly due to the flexibility and control they have over their work environment, combined with the financial pressure from not having worker’s compensation benefits. The social security system may influence the duration of sick leave, as receiving worker’s compensation has been linked to extended sick leave periods and lower rates of patients returning-to-work.^12,35,36^ Our research did not investigate the impact of worker’s compensation, yet it is commonly understood that people in self-employment often have less favorable worker’s compensation schemes.^12,35,36^ This may lead to a stronger motivation to return-to-work quicker in people who are self-employed.

In addition, individuals with a higher educational level returned to work faster than those with a lower educational level, consistent with other studies in similar populations.^8,12,13,35^ It is supposed that patients with a higher level of education tend to show a more profound understanding of disease processes, which results in more realistic expectations, treatment compliance, and therefore faster recoveries in both lumbar disc herniation and general populations.^37,38^ Furthermore, individuals with higher educational backgrounds are likely to have professions that demand less physical exertion, which may facilitate a faster return-to-work.^8^

Similarly, engaging in less physically demanding work was associated with a faster return-to-work time. In addition to clinical and demographic characteristics, job-related factors—such as employment type, physical demands, and perceived prognosis—have also been shown to influence return-to-work following lumbar discectomy.^39^ Despite ongoing debates regarding the impact of physically demanding jobs on the return-to-work process, the established association between job strenuousness and key recovery indicators, such as work resumption and sick leave duration, highlights the vital role of preoperative job functions in recovery planning.^11,12,22,36,40^

Higher disability scores, however, were linked to slower return-to-work times. Previous studies have linked higher baseline pain and disability scores with poorer outcomes following lumbar microdiscectomy, and these scores are widely recognized as prognostic indicators for musculoskeletal pain.^13,22,41,42^

Including the confounders, age, gender, and leg pain intensity, in our univariable and multivariable models did not significantly alter the findings, indicating that these factors are not crucial in determining the return-to-work time following lumbar microdiscectomy and subsequent physiotherapy. This suggests that the main factors studied—such as education level, employment status, disability levels, and physical work demands, have a more direct influence on recovery outcomes, highlighting their importance in both clinical assessment and rehabilitation.

This study has several limitations. First, we relied on routinely collected preoperative baseline data to explore associations with return-to-work. These data were collected prospectively using validated questionnaires as part of standard clinical care and were managed through a secure, automated PROMS system. This ensures consistent data collection across patients and reflects real-world clinical practice. While this method strengthens the generalizability and feasibility of our findings, we acknowledge that other potentially relevant factors, such as chronic conditions, psychological readiness, preoperative depression scores, opioid use, workplace accommodations, sitting hours, and job satisfaction, were not captured in this study and may also influence return-to-work outcomes.^12–16^ Identifying and incorporating these variables could provide a more comprehensive understanding of the factors influencing return-to-work.

Second, in our study, we used the questions “After how many weeks did you fully resume your work?” and “Do you currently have work currently” as outcome measure for returning-to-work. Comparing our results with other studies is challenging due to the absence of a standardized method for assessing returning-to-work.^12^ Research lacks a universally accepted measure for evaluating returning-to-work rates and the duration of sick leave, despite their importance as outcome measures for both societal and patient-centric evaluations.^12^

Third, we a priori classified patients who resumed work in the same role as having “fully returned to work,” while those who returned to their original employer but transitioned to a different role or performed modified tasks (*e.g.* less physically demanding duties) were classified as “not fully returned to work.” However, some patients may achieve a positive and sustainable outcome by transitioning to a different role. Given the small number of patients who changed roles in our study, their inclusion did not influence our main findings. Future qualitative studies should explore reasons why some patients choose to transition to a different role after surgery. In addition, it is important to acknowledge that patients not in paid employment before surgery were excluded from our analyses. Unpaid activities (*e.g.* household tasks), which may also represent meaningful functional recovery, were not evaluated. This should be considered when interpreting our results.

Fourth, a lack of Dutch proficiency was an exclusion criterion because all outcome measures were in Dutch. This may have limited the generalizability of our findings.

Fifth, although nearly all patients in our cohort (93.4%) received postoperative physiotherapy, with a median treatment duration of 14 weeks, the content, frequency, total number of sessions, and intensity of therapy varied. While all patients received the standardized rehabilitation protocol, treatment was tailored to individual goals, occupational demands, and clinical progress. This variation reflects real-world clinical practice and enhances generalizability. The exact number of physiotherapy sessions was, however, not consistently recorded. Future research should aim to identify the role of physiotherapy related to return-to-work outcomes more specifically.

Lastly, this study focused specifically on patients undergoing lumbar microdiscectomy, which is the standard surgical approach for lumbar radiculopathy in our national health care context.^43,44^ Our findings, therefore, apply to microdiscectomy and should not be generalized to other surgical techniques or patient populations without caution.

Collectively, these findings underscore the importance of providing patients with clear insights into expected return-to-work timelines based on individual factors such as job demands and personal circumstances. This approach can help set realistic goals and manage expectations, ultimately supporting a smoother recovery process.

## CONCLUSION

Overall, two-thirds of patients fully resumed their paid job in their original role with the same physical demands and responsibilities by 52 weeks. Our study underscores the complex interplay of work-related and personal factors influencing return-to-work.

Key Points

- Among patients who underwent lumbar microdiscectomy followed by postoperative physiotherapy for painful lumbar radiculopathy, 69.3% fully resumed their paid job in their original role with the same physical demands and responsibilities by 52 weeks.
- Some patients transitioned to different roles, including moving to less physically demanding positions, modifying tasks within their existing role, or taking on jobs with similar physical demands but different responsibilities.
- The median (IQR) return-to-work time was 16.0 weeks (14.0–16.0), with an 85.0% probability of returning-to-work within 26 weeks postsurgery.
- The likelihood of returning-to-work is significantly associated with higher education, self-employment, and the absence of physical work demands, while high disability scores decrease the likelihood of returning-to-work.
- Recognizing these factors can aid clinicians and patients in setting realistic expectations, establishing achievable goals, and promoting the patient’s return to preoperative function and employment.

## Supplementary Material
