# Supplementary material for: Timelines and Associated Factors for Return-to-Work of Patients With Painful Lumbar Radiculopathy Who Undergo Lumbar Microdiscectomy Followed by Physiotherapy: A Prospective Cohort Study
Source: Spine (Phila Pa 1976). 2025 Jul 14;50(23):1673–81. doi: 10.1097/BRS.0000000000005443 (PMC12594137; doi:10.1097/BRS.0000000000005443)
Supplement: Supplementary file 1 [file brs-50-1673-s001.docx]

**Appendix A:**

**Rehabilitation protocol: Physiotherapy after lumbar microdiscectomy**

**Introduction**:

Recovery after disc surgery varies individually, and is influenced by factors such as the duration of preoperative symptoms, the nature of back and leg pain, wound healing and the patient's overall physical condition. This protocol outlines a general guideline for physiotherapy following microdiscectomy for lumbar radiculopathy.

**Early post-operative phase**:

Schedule the first appointment as soon as possible post-discharge to provide the patient with lifestyle guidelines and feedback.

**Phase I (Week 1 to 4)**: Gradual increase in ADL (Activities of Daily Living) guided by pain tolerance.

- Educate patient on recovery and prognosis.
- Identify patient-specific, relevant and achievable activities and skills.
- Provide insight into load management in conjunction with lifestyle guidelines.
- Exercises for posture and movement awareness.
- Functional training.
- Begin enhancing overall physical condition.

**Phase II (Week 4 to 12)**: Resumption of ADL and progression to specific (work-related) skills.

- Support the patient in fully resuming ADL, through information, advice and instructions.
- Check and if needed improve joint, muscle and nerve health.
- Exercise therapy focusing on: strengthening (isometric) trunk muscles and lower extremity muscles, improving functional postures and movements, coordination of posture and movement.
- Increase overall load capacity.
- Educate on preventing future recurrence of symptoms.

**Phase III (3 to 6-months)**: Return to work and sports.

- Expand exercise therapy, now focusing more on work and sports-related activities, moving beyond isometric exercises if possible.
- Reinforce specific work and sports-related movement patterns.
- Enhance endurance and load-bearing training.
- Emphasize behavioural aspects: awareness and behavioral changes are often essential to prevent recurrences.
- The goal at the end of this phase is complete return to work and sports.

The protocol is designed to be adaptable based on individual patient needs and progress, emphasizing the importance of a patient-centered approach in rehabilitation.
