# Supplementary figures and images for: Timelines and Associated Factors for Return-to-Work of Patients With Painful Lumbar Radiculopathy Who Undergo Lumbar Microdiscectomy Followed by Physiotherapy: A Prospective Cohort Study
Source: Spine (Phila Pa 1976). 2025 Jul 14;50(23):1673–81. doi: 10.1097/BRS.0000000000005443 (PMC12594137; doi:10.1097/BRS.0000000000005443)

**Appendix B**


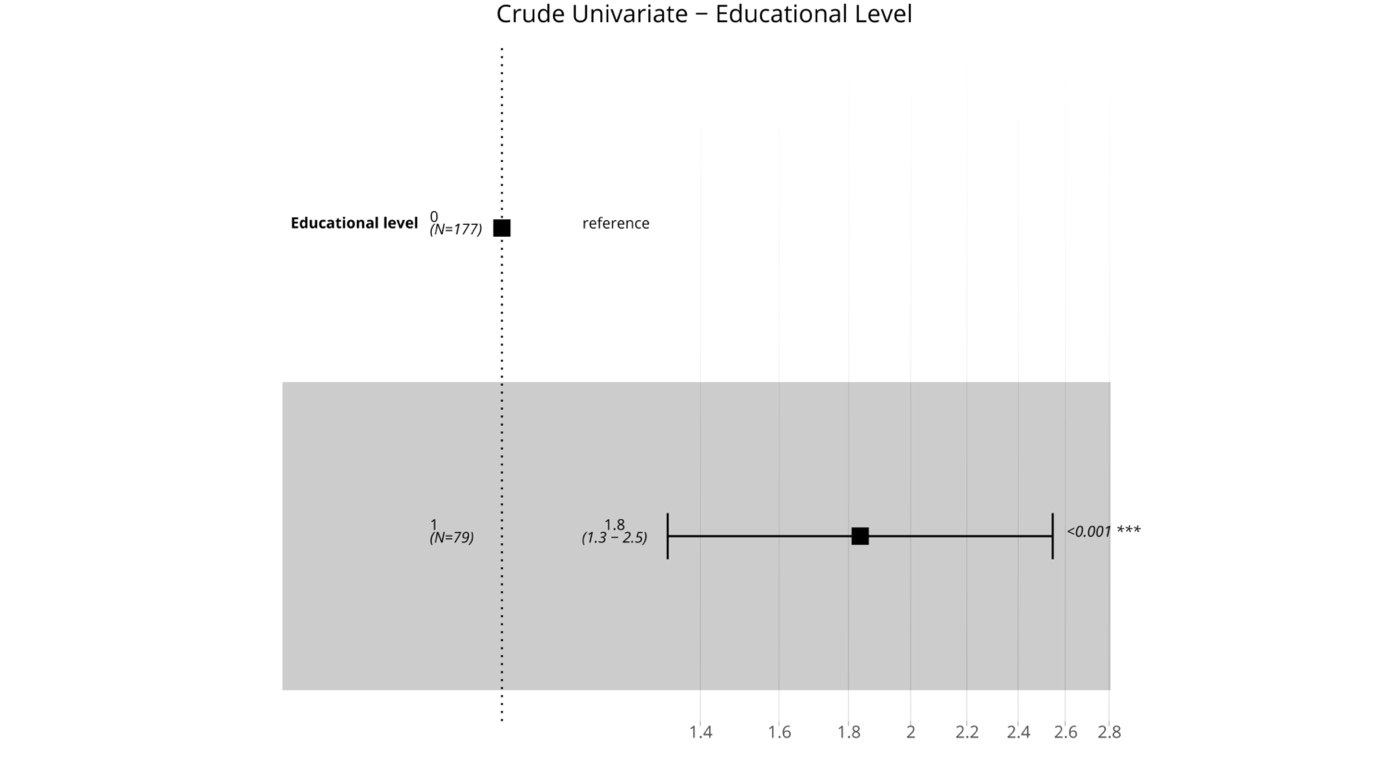


**Figure B1**.


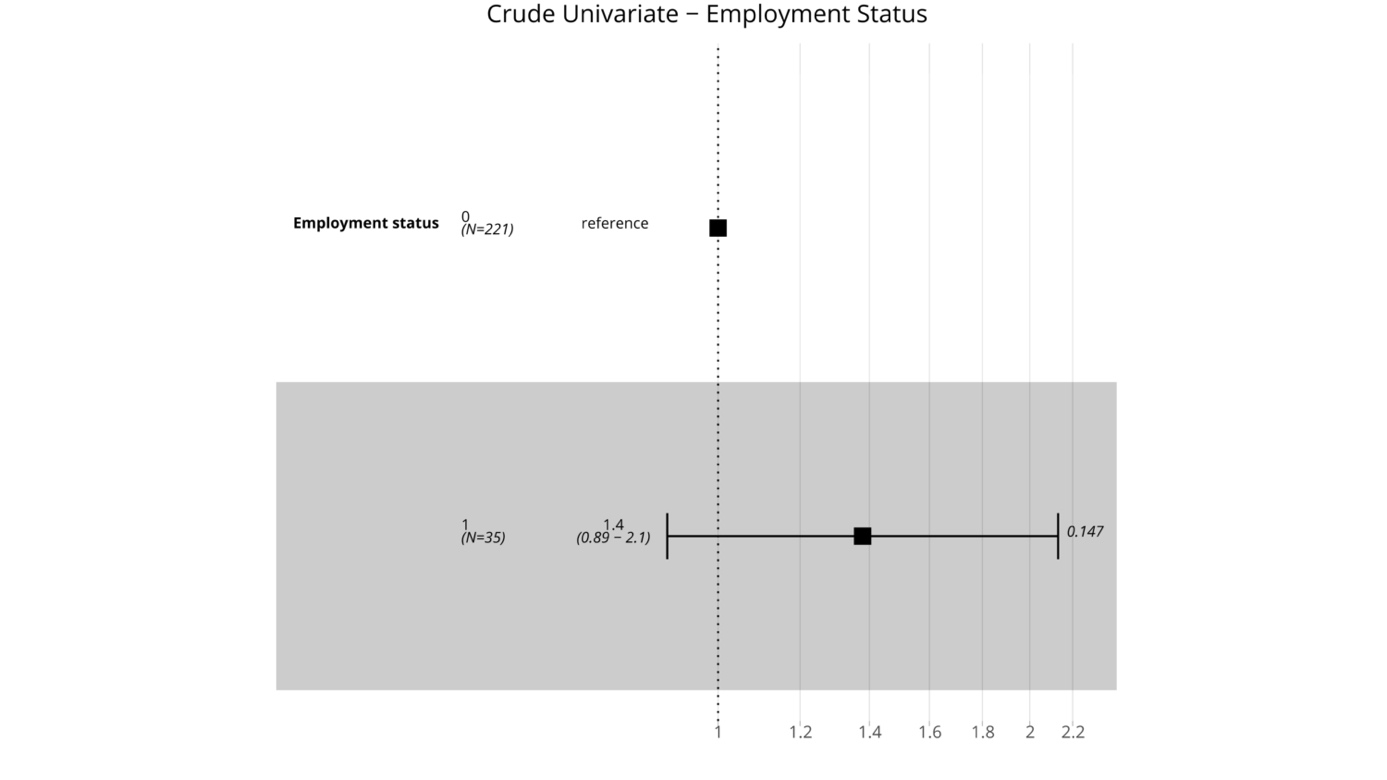


**Figure B2**.


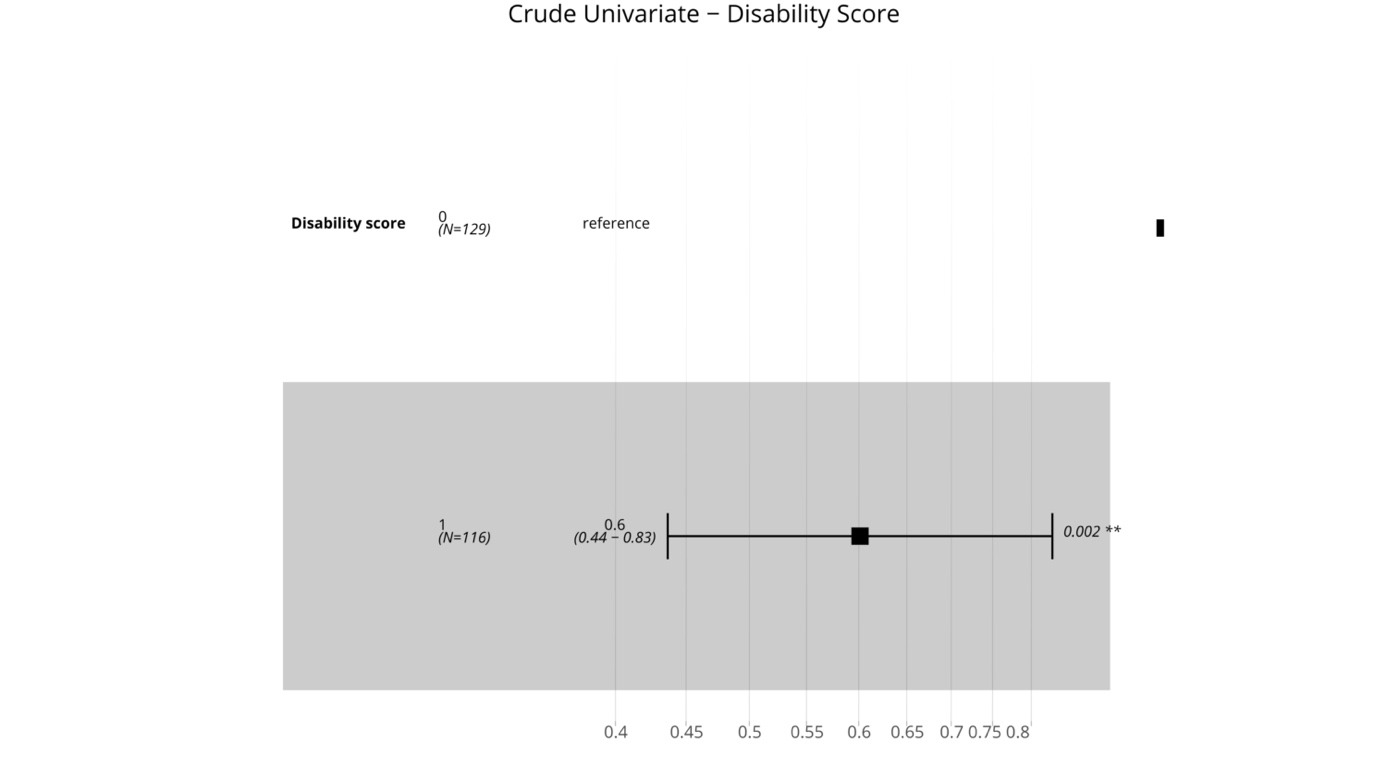


**Figure B3**.


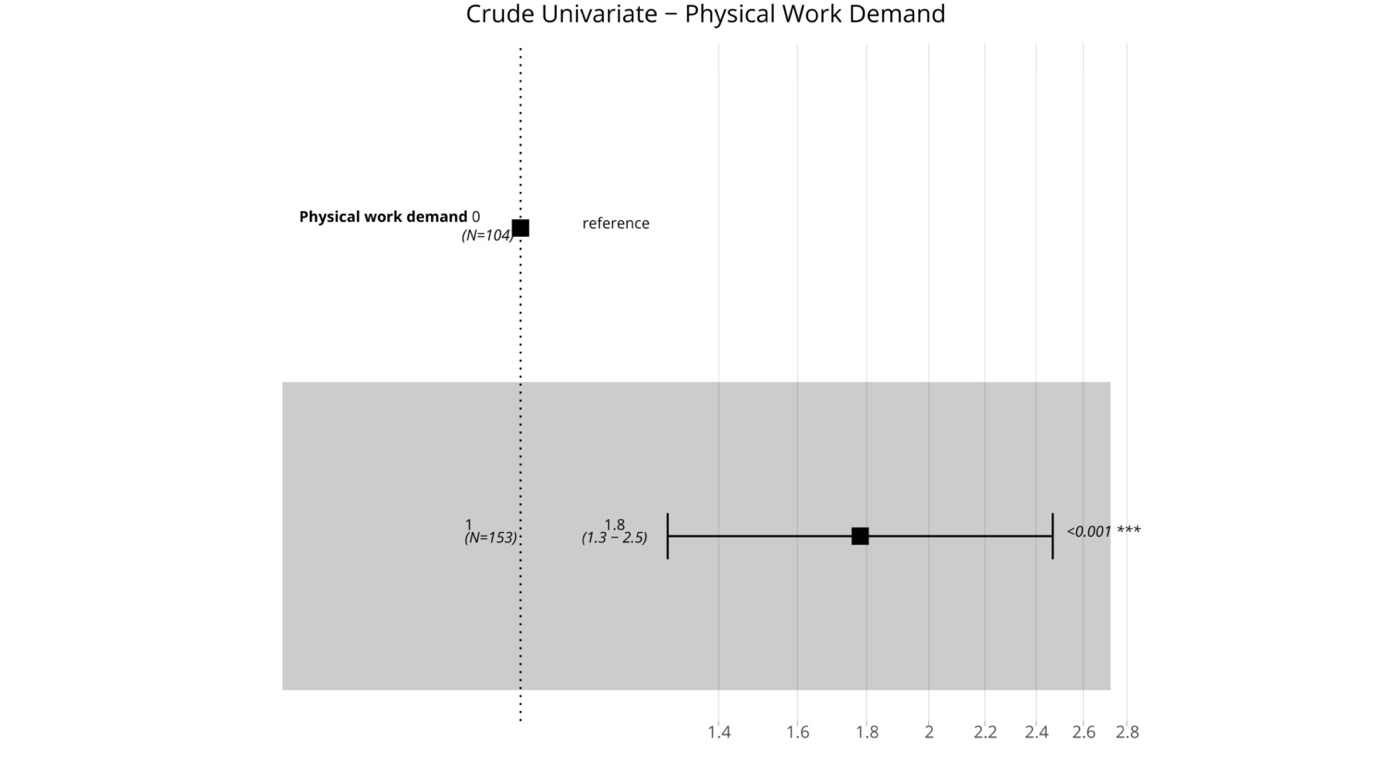


**Figure B4**.
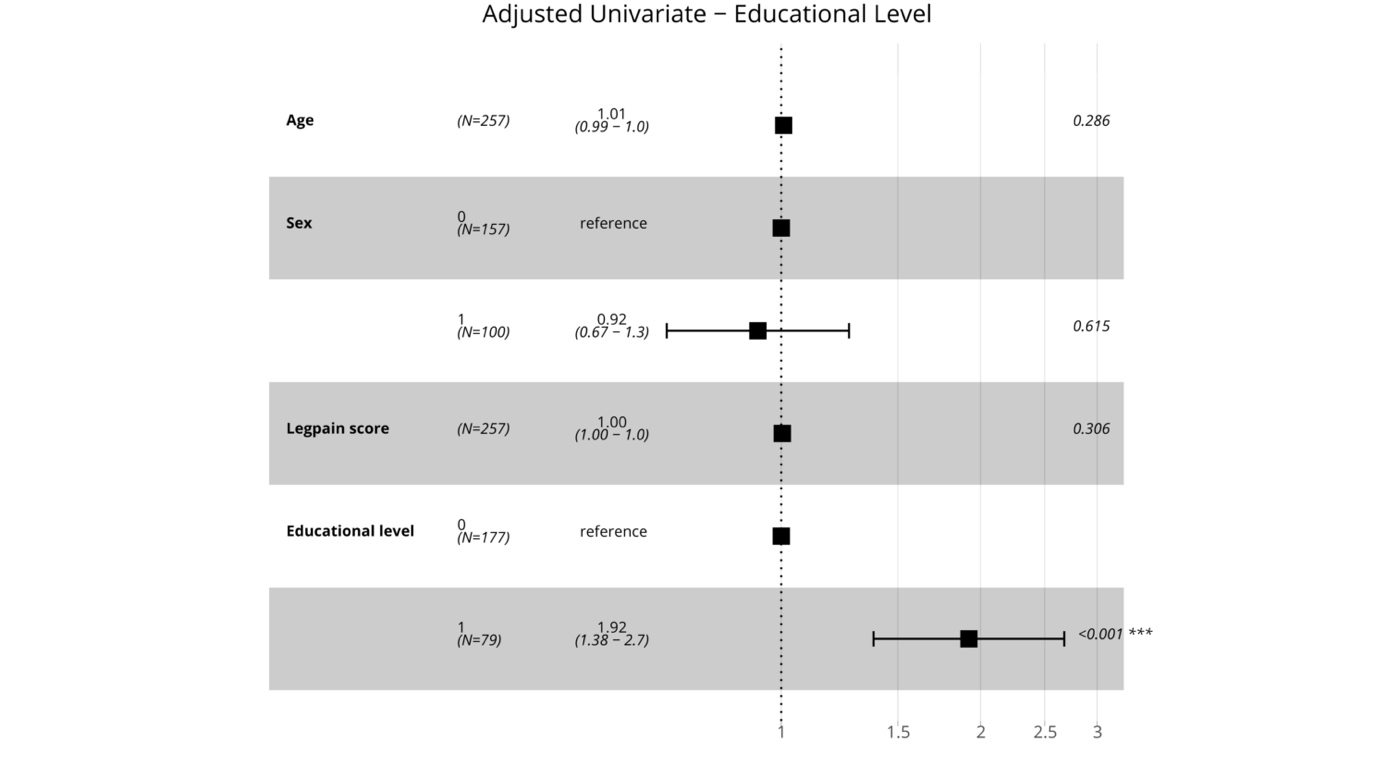


**Figure B5**.


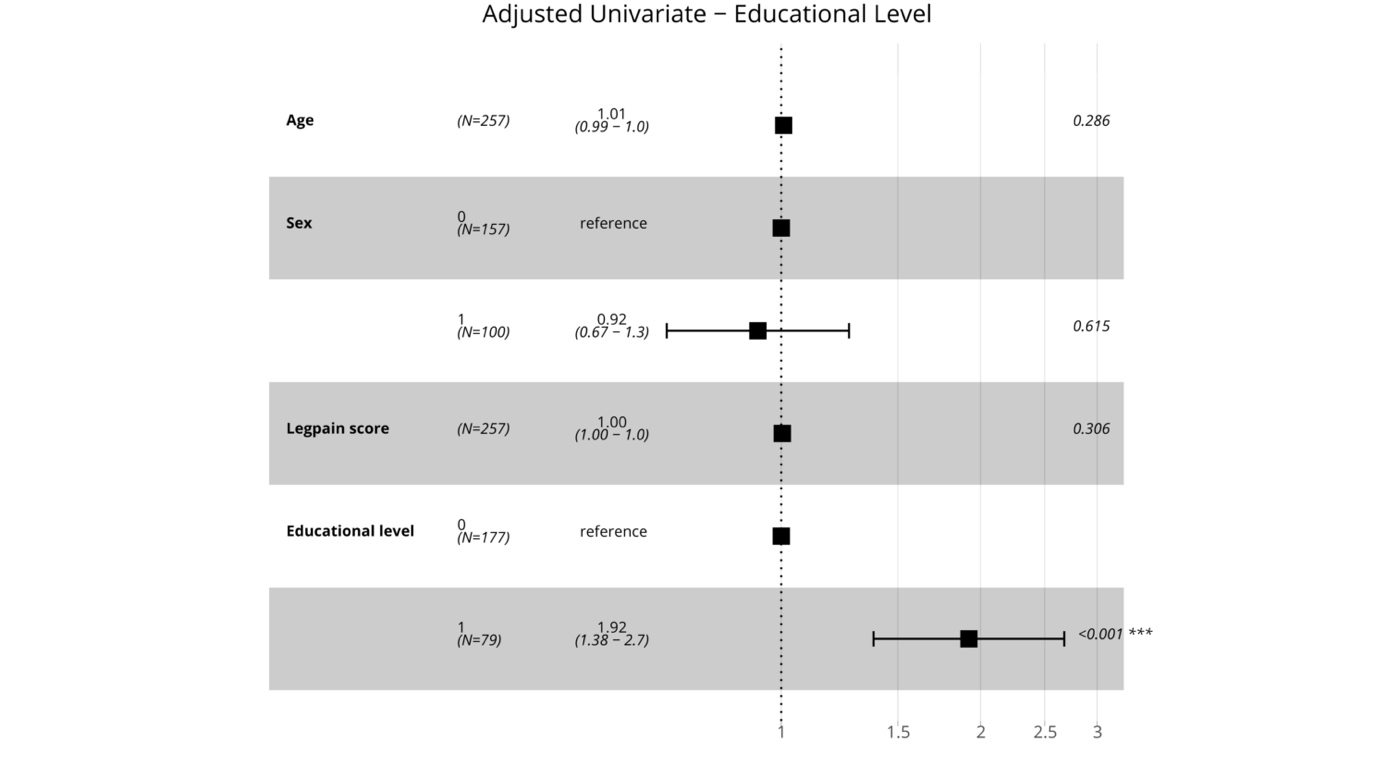


**Figure B6**.


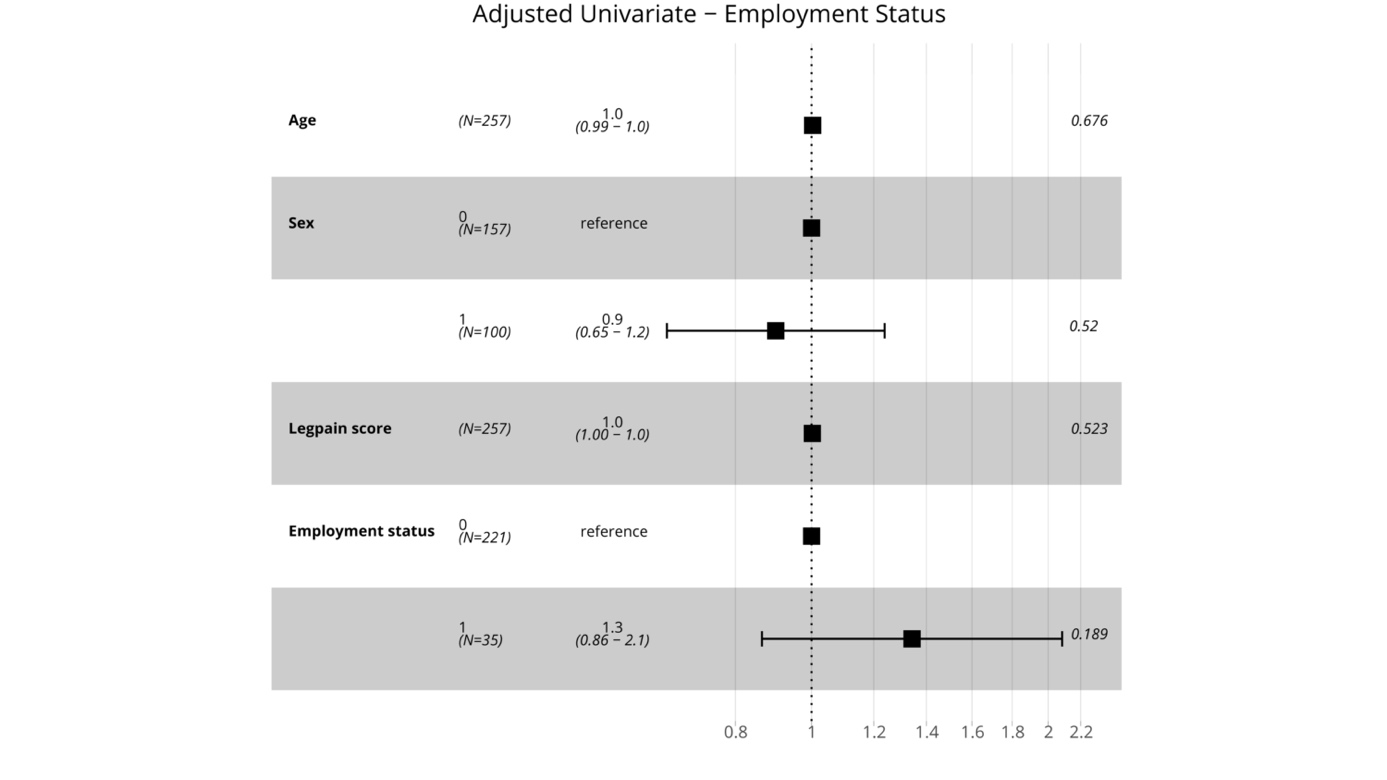


**Figure B7**.


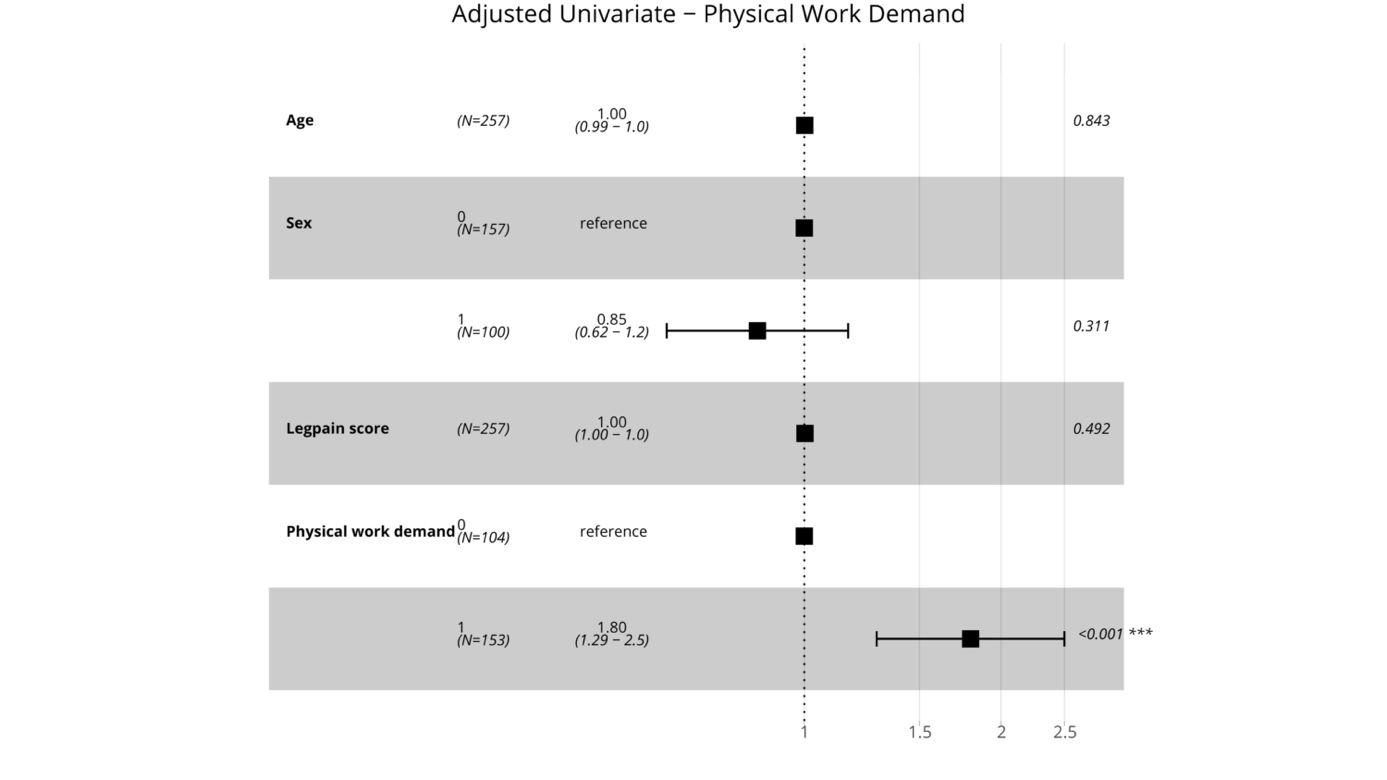


**Figure B8**.

Supplement: Supplementary file 2 [file brs-50-1673-s002.docx]

**Appendix C**


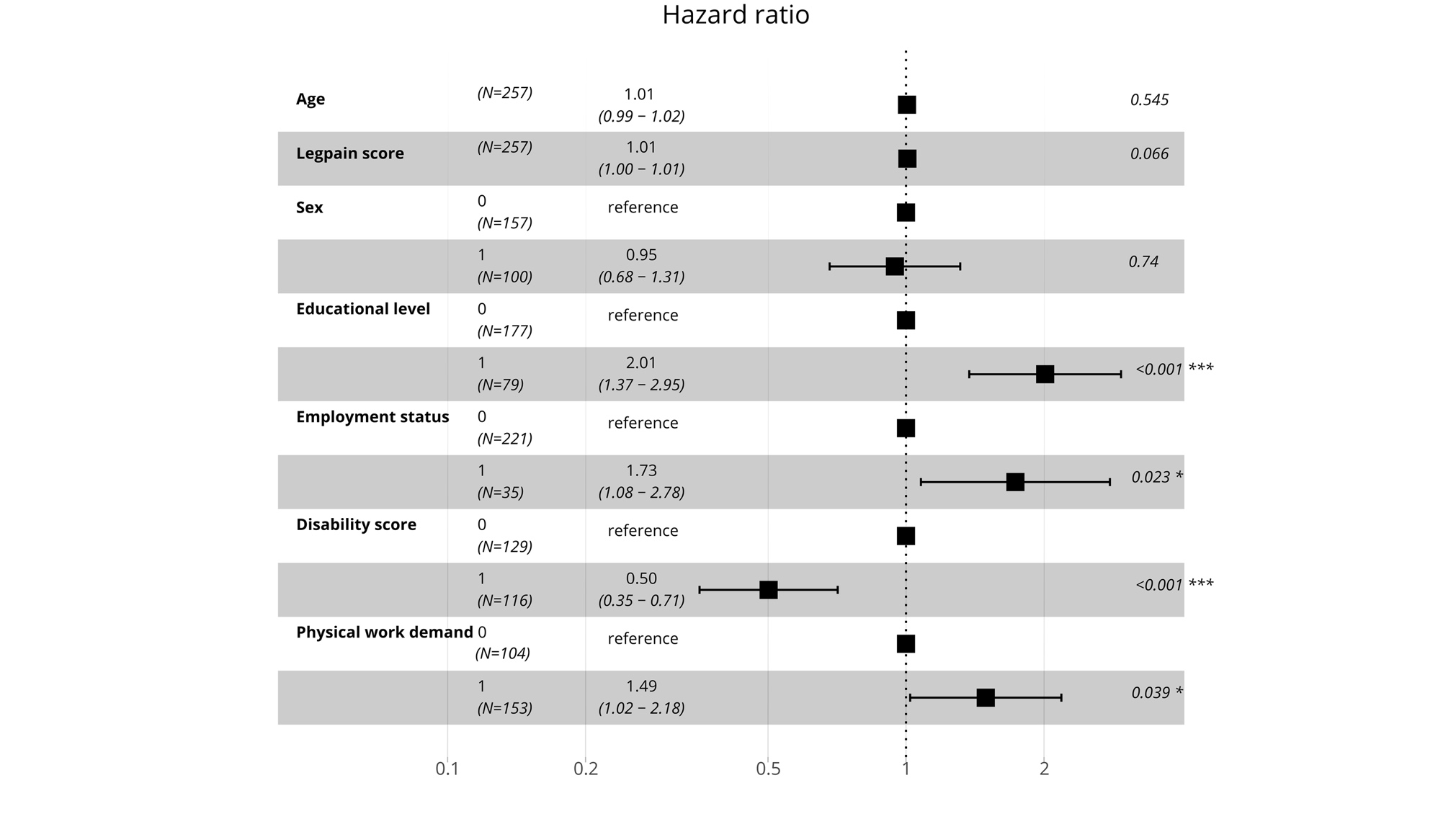


**Figure C1**.

Supplement: Supplementary file 3 [file brs-50-1673-s003.docx]
